# Has Public Interest in Elective Spine Surgery Returned to Pre-COVID 19 Levels? A Google Trends Analysis

**DOI:** 10.7759/cureus.22858

**Published:** 2022-03-04

**Authors:** Christopher R Michel, Christopher Dijanic, Suleiman Sudah, Daniel Kerrigan, Jason Cohen

**Affiliations:** 1 Orthopaedic Surgery, Monmouth Medical Center, Long Branch, USA; 2 Orthopedic Surgery, Monmouth Medical Center, Long Branch, USA

**Keywords:** posterior lumbar fusion, anterior cervical discectomy, pandemic, trends, google, surgery, covid-19, lumbar fusion, cervical fusion, public interest

## Abstract

Objective

To utilize Google trends to examine the effects of changing rules and regulations on public interest regarding elective spine surgery.

Methods

This is a retrospective review analyzing data from Google trends to quantify public interest in elective cervical and lumbar fusion as restrictions related to COVID-19 were released. Three time periods were created surrounding the release of restriction on elective surgery on March 13, 2020, by the Centers for Medicare and Medicaid Services (CMS). “Pre-COVID” was defined as the four-month period directly preceding the national ban on elective surgery (11/13/2019 to 3/13/2020). “COVID” was defined as the four-month time period directly after the national ban on elective surgery (3/13/20-7/13/20), and “Post-COVID” was defined as the time period starting four months after the restrictions on elective surgeries first took place (7/13/20-11/13/20). Relative search volume (RSV) was assessed during all three time periods and compared using an analysis of variance test.

Results

Search volume for all terms pertaining to cervical and lumbar fusion declined precipitously after the release of restrictions on elective surgery. Additionally, search volume has yet to return to pre-pandemic levels. However, for many of the terms public interest has been steadily increasing and signals the return in demand for these procedures.

Conclusion

Public interest in elective spine surgery has been increasing as restrictions continue to loosen and many patients that deferred care will drive increased demand for the foreseeable future.

## Introduction

The COVID-19 pandemic has presented unprecedented issues for patients and the healthcare community as a whole. During the pandemic, there has been a sharp shift in the demand for elective orthopedic procedures [1]. Typically, the demand for these elective orthopedic procedures is strongly correlated with both the health of the economy and the stock market [2]. However, on March 13, 2020, the president of the United States declared COVID-19 to be a national emergency, and the Centers for Medicare and Medicaid Services (CMS) followed shortly thereafter with restrictions on elective procedures [3,4]. As a result, a recommendation for healthcare facilities and surgical practices to “delay all elective surgeries, non-essential medical, surgical and dental procedures” was implemented. On 2 April 2020, the American Academy of Orthopedic Surgeons followed by releasing a statement recommending all elective orthopedic surgeries be canceled or postponed until further notice [5]. 

These recommendations combined with a lack of coveted resources including ICU beds, ventilators, and personal protective equipment caused many hospitals and health care systems across the nation to cease normal operations as priorities shifted to devoting staff and supplies to treat patients afflicted by the coronavirus. The restrictions placed on elective procedures, outpatient surgery centers, and hospitals alike created a unique situation in which public access to elective orthopedic procedures ceased overnight during the height of the pandemic. 

With decreased access to physicians, many patients suffering from spinal conditions turned to the internet. Internet usage rates among patients considering elective orthopedic procedures have been previously measured as high as 84% with up to 80% of these patients researching their condition online. Furthermore, 30% of these patients specifically discuss information found online with their surgeon [6-9]. . For spine specifically, studies have shown that one in four patients seen in the outpatient clinic has used the internet to research their spinal condition [10]. 

Google is by far the most commonly used search engine on the internet [11]. Google uses deep learning capability to condense millions of worldwide search queries into quantifiable measures of search volume. Google trend is a free tool that allows users to measure this volume over time for specific keywords [12]. It has been used in both short and long-term studies to evaluate the impact of specific events on interest regarding procedures in orthopedic surgery [13]. As restrictions loosen and elective orthopedic spine surgeries begin to resume, Google trends present a unique tool to gauge public interest over the time surrounding COVID-19 related restrictions which continue to evolve.

In this study, we utilize Google trends to examine the effects of changing rules and regulations on public interest regarding elective spine surgery. We hypothesize that, after initially declining as a result of covid-related restrictions and regulations surrounding elective surgery, public interest in elective spine procedures has returned and, in some cases exceeded, previous levels.

## Materials and methods

Two main groups were evaluated in this study: cervical fusion and lumbar fusion. Search terms were selected using the ‘related queries’ that are part of google trends. The three highest volume search terms pertaining to cervical and lumbar spine surgery were selected. The search terms ‘cervical fusion’, ‘cervical fusion surgery’ and ‘cervical spinal fusion’ for the cervical fusion group and ‘lumbar fusion’, ‘lumbar fusion surgery', ‘lumbar spinal fusion’ for the lumbar fusion group were entered on google trends. Search volume trends specifically for the United States for each of these groups were evaluated for three distinct time periods surrounding the COVID-19 shutdown of elective procedures. “Pre-COVID” was defined as the four-month period directly preceding the national ban on elective surgery (11/13/2019 to 3/13/2020). “COVID” was defined as the four-month time period directly after the national ban on elective surgery (3/13/20-7/13/20), and “Post-COVID” was defined as the time period starting four months after the restrictions on elective surgeries first took place (7/13/20-11/13/20). 

Our results were focused on search volume trends within the United States specifically, as CMS and the American Academy of Orthopedic Surgeons (AAOS) recommendations originated from this country. 

By entering these keywords into Google trends a concise graphic representation of search volume is presented for the specific time period requested. This interactive graph was then used to determine the peak search volume, which is assigned a relative search volume (RSV) value of 100-meaning that 100% of the peak volume was searched at that time. All other data points are reported as a percentage of this peak search volume. The scale of RSV is 0 to 100. Using these values, we were able to assess the increase or decrease in search volume over time relative to our three time periods. We also assessed the average RSV for the three time periods previously described. In addition, we assessed whether the difference in the average RSV for each time period was statistically significant using an analysis of variance test (Microsoft Excel, Redmond, WA). 

## Results

**Figure 1:**
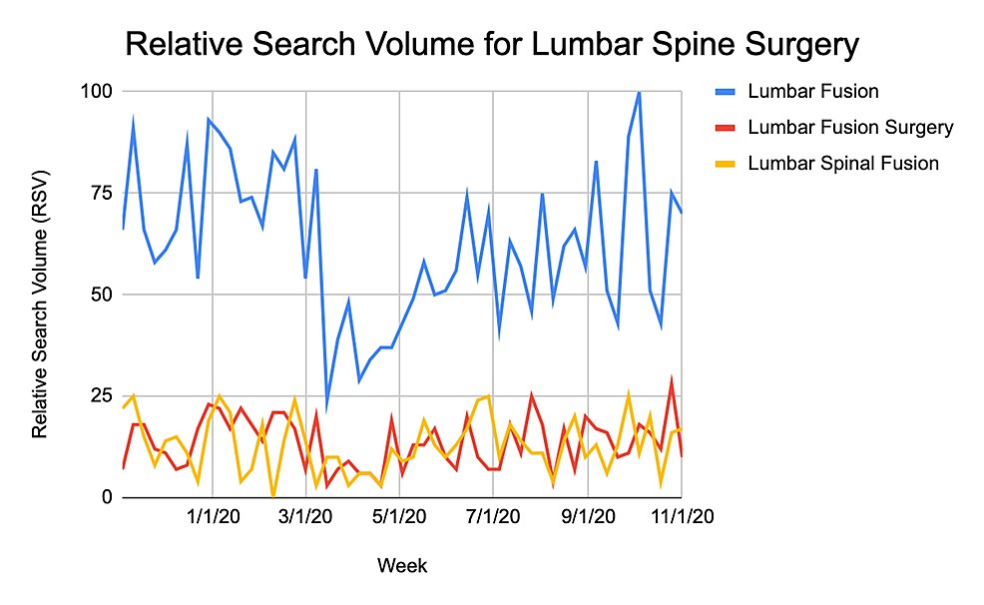
Relative search volume for three terms related to lumbar spine surgery over the course of all three time periods

When all three phrases pertaining to lumbar spine surgery (‘lumbar fusion’, ‘lumbar fusion surgery’, and ‘lumbar spinal fusion’) were compared, ‘lumbar fusion’ showed consistently higher relative search volume (RSV) compared to the other two during all time periods. The mean RSV for the three search phrases relative to one another over all three time periods were 62.2 +/- 18.3, 13.6 +- 6.2, and 13.01 +/- 6.7 for ‘lumbar fusion’, ‘lumbar fusion surgery’, and ‘lumbar spinal fusion’ respectively as shown in Table 1. The difference in RSV for ‘lumbar fusion’ was found to be statistically significant for the three distinct time periods with p=0.000236. The RSV for all three search terms is plotted over time as shown in Figure 1.

**Table 1:** Week by week comparison of relative search volume (RSV) for lumbar spine surgery during the three time periods surrounding the release of COVID-19 related restrictions on elective procedures.

Week	Lumbar Fusion	Lumbar Fusion Surgery	Lumbar Spinal Fusion
11/3/19	66	7	22
11/10/19	91	18	25
11/17/19	66	18	15
11/24/19	58	12	8
12/1/19	61	11	14
12/8/19	66	7	15
12/15/19	87	8	11
12/22/19	54	17	4
12/29/19	93	23	19
1/5/20	90	22	25
1/12/20	86	17	21
1/19/20	73	22	4
1/26/20	74	18	7
2/2/20	67	14	18
2/9/20	85	21	0
2/16/20	81	21	14
2/23/20	88	17	24
3/1/20	54	7	14
3/8/20	81	20	3
3/15/20	24	3	10
3/22/20	39	7	10
3/29/20	48	9	3
4/5/20	29	6	6
4/12/20	34	6	6
4/19/20	37	3	3
4/26/20	37	19	12
5/3/20	43	6	9
5/10/20	49	13	10
5/17/20	58	13	19
5/24/20	50	17	13
5/31/20	51	10	10
6/7/20	56	7	13
6/14/20	74	20	17
6/21/20	55	10	24
6/28/20	70	7	25
7/5/20	42	7	10
7/12/20	63	18	18
7/19/20	57	11	14
7/26/20	46	25	11
8/2/20	75	18	11
8/9/20	49	4	4
8/16/20	62	17	14
8/23/20	66	7	20
8/30/20	57	20	10
9/6/20	83	17	13
9/13/20	51	16	6
9/20/20	43	10	13
9/27/20	89	11	25
10/4/20	100	18	11
10/11/20	51	16	20
10/18/20	43	12	4
10/25/20	75	28	16
11/1/20	70	10	17
Mean	62.20754717	13.60377358	13.01886792
Standard devation	18.32543261	6.193481755	6.68664158

**Figure 2:**
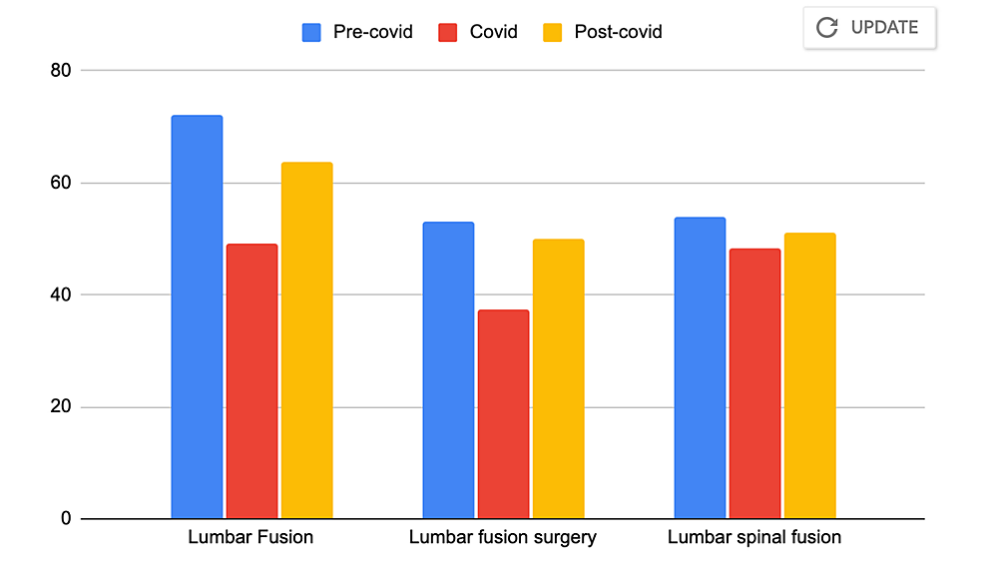
Mean relative search volume (RSV) for the three terms related to lumbar spine surgery compared over the three time periods

RSV for ‘lumbar fusion’ specifically was lowest on 3/15/2020 immediately after CMS and AAOS restrictions were placed on elective procedures. RSV for ‘lumbar fusion’ peaked on 10/4/2020 during the post-covid time period when restrictions began to be lifted. Similarly, RSV for ‘lumbar fusion surgery’ specifically was lowest on 4/19/2020, during the covid time period. RSV for this phrase peaked on 10/25/2020 during the post-covid time period. The mean RSV for the three search terms for each time period is shown in Table 2. When an analysis of variance was run, the only search term with a statistically significant difference between the three time periods was 'lumbar fusion' with p = .000236 as shown in Table 2. The mean RSV for each time period is plotted as a bar graph in Figure 2. 

**Table 2:** Average relative search volume (RSV) for lumbar spine surgery during the three time periods surrounding the release of COVID-19 related restrictions on elective procedures. An asterisk denotes statistical significance.

		Lumbar Fusion	Lumbar fusion surgery	Lumbar spinal fusion
11/13/19-3/13/20	Pre-covid	72.25	53	53.8
3/13/20-7/13/20	Covid	49.12	37.4	48.4
7/13/20-11/13/20	Post-covid	63.6	49.9	51.1
	P value	0.000236*	0.101783	0.82874

**Figure 3:**
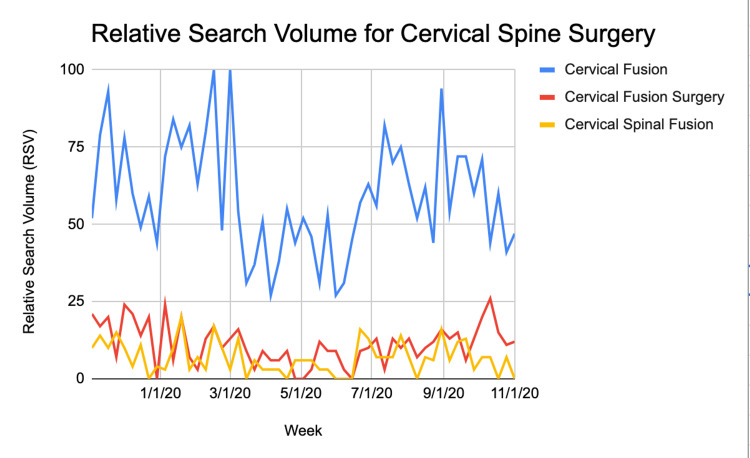
Relative search volume for three terms related to cervical spine surgery over the course of all three time periods

When all three phrases pertaining to cervical spine surgery (‘cervical fusion’, ‘cervical fusion surgery’, and ‘cervical spinal fusion’) were compared, ‘cervical fusion’ consistently showed higher RSV when compared to the other two phrases across all three time periods as shown in Table 3. These results are plotted over time in Figure 3.

**Table 3:** Week by week comparison of relative search volume (RSV) for cervical spine surgery during the three time periods surrounding the release of COVID-19 related restrictions on elective procedures.

Week	Cervical Fusion	Cervical Fusion Surgery	Cervical Spinal Fusion
11/3/19	52	21	10
11/10/19	79	17	14
11/17/19	93	20	10
11/24/19	58	7	15
12/1/19	78	24	10
12/8/19	60	21	4
12/15/19	49	14	11
12/22/19	59	20	0
12/29/19	44	0	4
1/5/20	72	24	3
1/12/20	84	6	10
1/19/20	75	20	20
1/26/20	82	7	3
2/2/20	63	3	7
2/9/20	80	13	3
2/16/20	100	17	17
2/23/20	48	10	10
3/1/20	100	13	3
3/8/20	54	16	13
3/15/20	31	9	0
3/22/20	37	3	6
3/29/20	51	9	3
4/5/20	27	6	3
4/12/20	38	6	3
4/19/20	55	9	0
4/26/20	44	0	6
5/3/20	52	0	6
5/10/20	46	3	6
5/17/20	31	12	3
5/24/20	53	9	3
5/31/20	27	9	0
6/7/20	31	3	0
6/14/20	45	0	0
6/21/20	57	9	16
6/28/20	63	10	13
7/5/20	56	13	7
7/12/20	82	3	7
7/19/20	70	13	7
7/26/20	75	10	14
8/2/20	63	13	7
8/9/20	52	7	0
8/16/20	62	10	7
8/23/20	44	12	6
8/30/20	94	16	16
9/6/20	54	13	6
9/13/20	72	15	12
9/20/20	72	6	13
9/27/20	60	13	3
10/4/20	71	20	7
10/11/20	44	26	7
10/18/20	60	15	0
10/25/20	41	11	7
11/1/20	47	12	0
Mean	59.18867925	11.28301887	6.811320755
Standard deviation	18.38483552	6.587801543	5.207452514

**Figure 4:**
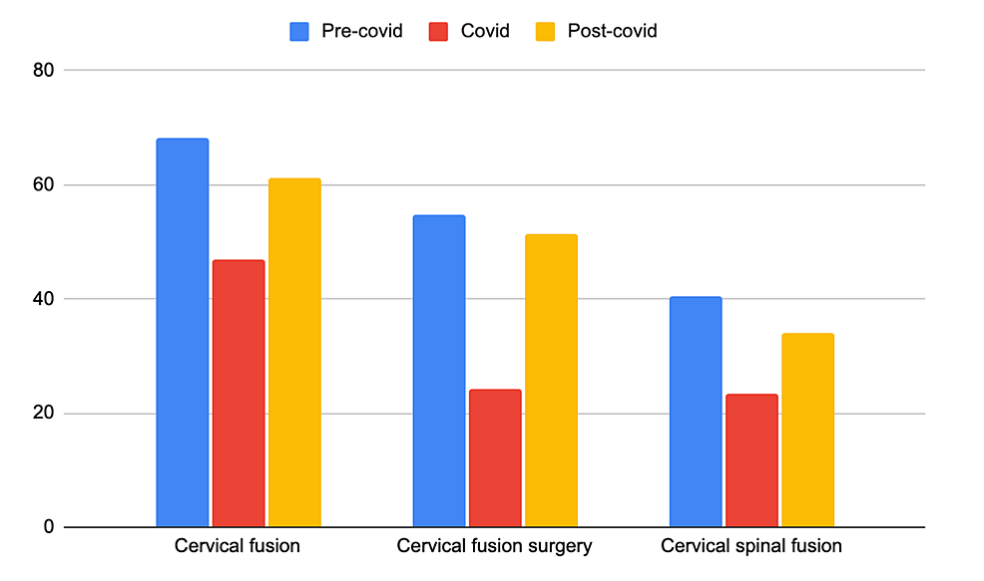
Mean relative search volume (RSV) for the three terms related to cervical spine surgery compared over the three time periods

RSV for ‘cervical fusion’ specifically was lowest on 4/5/20 during the covid period and highest during pre-covid on 2/16/20. RSV for ‘cervical fusion surgery’ was lowest on 4/26/2020 during the covid period and highest on 10/11/2020 in the post-covid period. Lastly, RSV for ‘cervical spinal fusion’ was lowest on 6/7/2020 during covid, and highest on 1/19/20 during the pre-covid period. The mean RSV for the three search terms for each time period is shown in Table 4. When an analysis of variance was run, the only search term with a statistically significant difference between the three time periods was 'cervical fusion' and 'cervical fusion surgery' with p=0.00096 and p=0.00015 respectively. The mean RSV for each time period is plotted as a bar graph in Figure 4.

**Table 4:** Average relative search volume (RSV) for cervical spine surgery during the three time periods surrounding the release of COVID-19 related restrictions on elective procedures. An asterisk denotes statistical significance.

	‘Cervical fusion’	‘Cervical fusion surgery’	‘Cervical spinal fusion’
Pre-Covid	68.1	54.8	40.5
Covid	46.8	24.1	23.4
Post-Covid	61.3	51.5	33.9
P value	.00096*	.00015*	.11422

## Discussion

The recommendations made by CMS and AAOS in March 2020 caused elective procedures to come to a screeching halt, creating an unprecedented situation. These recommendations not only postponed elective spine procedures, but forced orthopedic surgeons to adapt to a changing healthcare environment placing a heavier emphasis on telehealth visits as well as office-based procedures. In addition, more telehealth visits have led patients to seek information from internet searches regarding spine surgery. This study suggests that public interest in elective spine surgery has returned to and in some cases exceeded pre-covid levels.

Based on our results, it is evident that search volumes for keywords pertaining to elective lumbar fusion declined precipitously surrounding the height of the pandemic in March 2020. For two out of three keywords, the lowest search volume occurred during the covid time period. The search volume for the third key phrase (‘lumbar spinal fusion’) was lowest in February 2020, approximately one month prior to the release of the CMS and AAOS recommendations. RSV for ‘lumbar fusion’, ‘lumbar fusion surgery’, and ‘lumbar spinal fusion’ all declined during the covid period by 23%, 15.6%, and 5.4% respectively. During the post-covid period, however, interest rebounded by 14.5%, 12.5%, and 2.7% respectively. Rebounding interest can most likely be attributed to patients deferring care during the pandemic. This increased interest, however, has yet to reach the level it was during the pre-covid period. Comparing pre-covid interest to post-covid, RSV declined 8.65%, 3.1%, and 2.7% for ‘lumbar fusion, ‘lumbar fusion surgery’, and ‘lumbar spinal fusion’ respectively. 

Similar trends are seen with the three terms related to cervical spine procedures we examined (‘cervical fusion’, ‘cervical fusion surgery’, and ‘cervical spinal fusion’). RSV declined for all three phrases from pre-covid to covid by 21%, 31%, and 17% for ‘cervical fusion’, ‘cervical fusion surgery’, and ‘cervical spinal fusion’ respectively. Interest returned as restrictions were lifted, showing 14%, 27%, and 10.5% increases from the covid period to post-covid for the three phases respectively. Like the terms related to lumbar fusion, however, interest in the terms related to cervical fusion has yet to return to pre-covid levels. Comparing pre-covid interest to post-covid, RSV declined 6.8%, 3.3%, and 6.6% for ‘cervical fusion, ‘cervical fusion surgery’, and ‘cervical spinal fusion’ respectively. 

Previous studies have demonstrated a significantly positive correlation between google trends and actual healthcare utilization [14]. This suggests that analysis of data obtained from google trends may prove useful in tracking public interest in spine procedures. Our study supports this, as search volume for all three search phrases showed an initial decrease in search volume from pre-covid to covid and an increase in RSV from the covid to post-covid periods as restrictions surrounding elective surgery began to be lifted [15]. As a result, telemedicine became a mainstay in the tools healthcare providers use to communicate with patients [16]. Despite this, nearly half of all adults reported that either they or someone in their household deferred medical care during the pandemic [17]. Furthermore, 30.2% of patients who delayed care were concerned about potential long-term health consequences, with needs centered around orthopedics and surgery [18]. As vaccines are distributed and healthcare organizations anticipate patient volumes for elective procedures returning to normal, Google trends represent a unique tool to anticipate this changing landscape and to meet increased demand.

There are several limitations to our study. User data and demographics are unfortunately not available for google trends. This demographic data would be helpful in assessing the types of patients seeking information regarding elective spine surgery over time. Additionally, google trends provide relative search volume as opposed to actual search volume. Lastly, there are additional search engines that people may use to find information on the internet including bing, yahoo, and duckduckgo. Unfortunately, data from these search engines are not publicly available at this time. We believe additional studies are warranted to assess the needs of patients who deferred care during the pandemic.

## Conclusions

This study illustrates the return of public interest in spine surgery surrounding the restrictions placed on elective procedures due to COVID 19. It is our belief that google trends can be a useful tool to the surgeon in gauging public interest in these procedures. This will allow healthcare systems to anticipate increasing patient volumes as COVID vaccines become more ubiquitous and more patients get vaccinated. By providing real-time insights into trends in the public interest, healthcare systems can allocate resources to meet the growing demand for these elective procedures. 

## References

[1] Diaz A, Sarac BA, Schoenbrunner AR, Janis JE, Pawlik TM (2020). Elective surgery in the time of COVID-19. Am J Surg.

[2] Agabiti N, Picciotto S, Cesaroni G (2007). The influence of socioeconomic status on utilization and outcomes of elective total hip replacement: a multicity population-based longitudinal study. Int J Qual Health Care.

[3] (2021). CMS releases recommendations on adult elective surgeries, non-essential medical, surgical, and dental procedures during COVID-19 response. https://www.cms.gov/newsroom/press-releases/cms-releases-recommendations-adult-elective-surgeries-non-essential-medical-surgical-and-dental.

[4] Adams DJM. Surgeon General (2021). Surgeon general: Delay elective medical, dental procedures to help us fight coronavirus. https://www.cms.gov/newsroom/press-releases/cms-releases-recommendations-adult-elective-surgeries-non-essential-medical-surgical-and-dental.

[5] (2021). AAOS guidelines for elective surgery during the COVID-19 pandemic. https://www.aaos.org/about/covid-19-information-for-our-members/aaos-guidelines-for-elective-surgery/.

[6] Fraval A, Ming Chong Y, Holcdorf D, Plunkett V, Tran P (2012). Internet use by orthopaedic outpatients - current trends and practices. Australas Med J.

[7] Baker JF, Green J, Synnott KA, Stephens MM, Poynton AR, Mulhall KJ (2013). Internet use in an orthopaedic outpatient population. Curr Orthop Pract.

[8] Sechrest RC (2010). The internet and the physician-patient relationship. Clin Orthop Relat Res.

[9] Cassidy JT, Baker JF (2016). Orthopaedic patient information on the world wide web: an essential review. J Bone Joint Surg Am.

[10] Baker JF, Devitt BM, Kiely PD, Green J, Mulhall KJ, Synnott KA, Poynton AR (2010). Prevalence of internet use amongst an elective spinal surgery outpatient population. Eur Spine J.

[11] (2021). Search engine market share worldwide. https://gs.statcounter.com/search-engine-market-share.

[12] (2021). The homepage explained. https://support.google.com/trends/answer/6248105.

[13] Tijerina JD, Cohen SA, Parham MJ, Debbaut C, Cohen L, Stevanovic M, Lefebvre R (2020). Public interest in elective orthopedic surgery following recommendations during COVID-19: a google trends analysis. Cureus.

[14] Tijerina JD, Morrison SD, Nolan IT, Parham MJ, Nazerali R (2020). Predicting public interest in nonsurgical cosmetic procedures using google trends. Aesthet Surg J.

[15] (2021). State resumption of elective surgery orders, guidance, and resources. June 5 O.

[16] Wosik J, Fudim M, Cameron B (2020). Telehealth transformation: COVID-19 and the rise of virtual care. J Am Med Inform Assoc.

[17] KFF Health Tracking Poll - May 2020. KFF (2021). KFF Health Tracking Poll - May 2020. https://www.kff.org/coronavirus-covid-19/report/kff-health-tracking-poll-may-2020/.

[18] Atherly A, Van Den Broek-Altenburg E, Hart V, Gleason K, Carney J (2020). Consumer reported care deferrals due to the COVID-19 pandemic, and the role and potential of telemedicine: cross-sectional analysis. JMIR Public Health Surveill.

